# Urgent endovascular treatment of juxtarenal aneurysm using a parallel grafts technique

**DOI:** 10.1590/1677-5449.202000282

**Published:** 2024-10-04

**Authors:** Fábio Augusto Cypreste Oliveira, Bruno Campos Coelho, Marina Balestra, Juliana Caetano Barreto Cypreste Oliveira, Maria Cunha Ribeiro Amorelli, Davi Douglas Heckmann, Carlos Eduardo de Sousa Amorelli, Fábio Lemos Campedelli

**Affiliations:** 1 Hospital São Francisco de Assis, Serviço de Cirurgia Vascular e Endovascular, Goiânia, GO, Brasil.; 2 Universidade Federal de Goiás – UFG, Hospital das Clínicas, Angiorradiologia e Cirurgia Endovascular, Goiânia, GO, Brasil.; 3 Universidade Federal de Goiás – UFG, Hospital das Clínicas, Infectologia, Goiânia, GO, Brasil.; 4 Hospital São Francisco de Assis, Hematologia e Hemoterapia, Goiânia, GO, Brasil.; 5 CenterVasc Rio, Rio de Janeiro, RJ, Brasil.

**Keywords:** abdominal aortic aneurysm, endovascular procedures, stents

## Abstract

Abdominal aortic aneurysms can constitute a therapeutic challenge in several anatomical scenarios, making endovascular treatment more complex. A juxtarenal abdominal aortic aneurysm (JR-AAA) is defined by the absence of a proximal landing zone in the infrarenal segment and, therefore, techniques must be used to obtain an adequate neck for fixing the endoprostheses without provoking occlusion of renal arteries and visceral branches. The parallel grafts technique, physician-modified stent-graft, industry customized endoprostheses, and off-the-shelf branched endoprosthesis are techniques used in abdominal aneurysms with inadequate proximal neck, but each technique has its indications, limitations, and risks. In this therapeutic challenge, we present a case of urgent endovascular treatment of a JR-AAA using a parallel grafts technique, with good medium-term results, and discuss the therapeutic options.

## INTRODUCTION

Since it was first developed, at the start of the 1990s, endovascular repair of abdominal aortic aneurysms (EVAR) has evolved to a point at which more than 70% of all surgical abdominal aorta aneurysms can be repaired using this technique.^1^ However, difficulties related to approach routes, circumferential calcifications, extreme neck angles, and absent or inadequate proximal and/or distal necks can make EVAR a challenge.

Juxtarenal abdominal aortic aneurysm (JR-AAA) is characterized by aneurysmal dilatation starting immediately below the origin of the renal arteries, causing absence of a proximal landing zone in the infrarenal segment. In such cases, endovascular techniques can be used to obtain an adequate proximal neck to achieve proximal sealing without causing occlusion of the visceral branches of the abdominal aorta.^2^

Parallel stent grafting was originally introduced as a rescue maneuver for maintaining patency of branches of the aorta that had been inadvertently covered during EVAR.^3^ Parallel stenting techniques, such as chimney, snorkel, and sandwich grafts, offer lower cost options than customized devices (CMD) or off-the-shelf (OTS) options and are more likely to be used because of availability, making them an important option for emergency and urgent treatment of aortic aneurysms.

Initial experience with the snorkel technique was described in a 2003 publication by Greenberg et al.^4^ and Lobato and Camacho-Lobato^5^ described their experience with the sandwich technique in 2012.

These techniques appear to achieve good conformability between the main endoprosthesis, the visceral stent, and the wall of the aorta, but there is potential for leaks to develop between the devices in parallel causing a specific type of leak known as a gutter leak.^6^

Below, we report on a case of urgent treatment of a patient with a JR-AAA, using the chimney technique (Ch-EVAR) as first-choice treatment.

## PART I –CLINICAL SITUATION

A 62-year-old, hypertensive, male patient with a history of coronary angioplasty performed 2 years previously was admitted with intense abdominal pains, radiating to the back, with sudden and progressive onset 24 hours earlier. Physical examination found him hemodynamically stable, with a distended abdomen that was painful on deep palpation, but with no signs of peritoneal irritation and normal pulses in both lower limbs.

**Figure 1:**
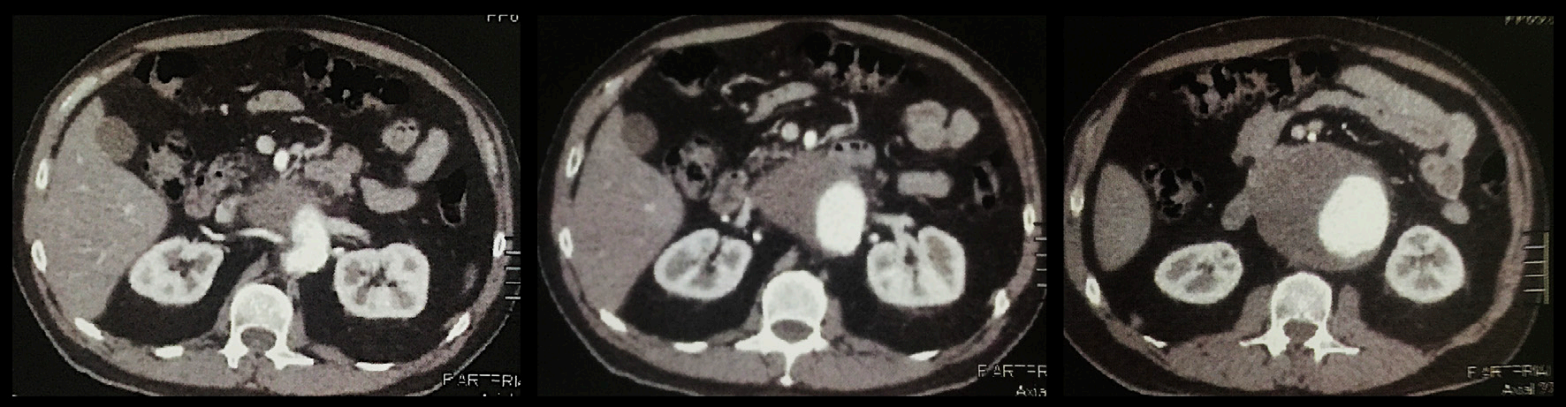
Axial angiotomography of the abdomen and pelvis with venous contrast showing a large volume juxtarenal aortic aneurysm.

Computed tomography with contrast of the abdomen and pelvis showed a JR-AAA with a maximum diameter of 82 mm and no other findings that would explain the abdominal pains (Figure 1). In view of the imminent risk of rupture and death, he was indicated for urgent surgical treatment, and the following possible surgical techniques applicable at the time were considered:

1- Conventional surgery with suprarenal clamping;
2- Endovascular treatment with a physician-modified stent;
3- Endovascular treatment with chimneys for both renal arteries.

## PART II – WHAT WAS DONE

Since the patient had a high cardiological risk rating — ASA III, according to the American Society of Anesthesiology classification^7^ — and in view of the need for suprarenal clamping, which increases surgical morbidity and risk of renal failure, the decision was taken to conduct endovascular treatment with the parallel stents technique.

A physician-modified stent graft was another option for endovascular treatment, but one that would increase the duration of surgery and the technical difficulty. Customization was not a feasible option because of the time needed for fabrication, sterilization, and delivery of the endoprosthesis. The possibility of thoracoabdominal branching was not considered because there was a healthy neck between the superior mesenteric artery and the start of the aneurysm (28 mm) and the diameter of the aorta at the level of the celiac trunk was less than 28 mm.

**Figure 2:**
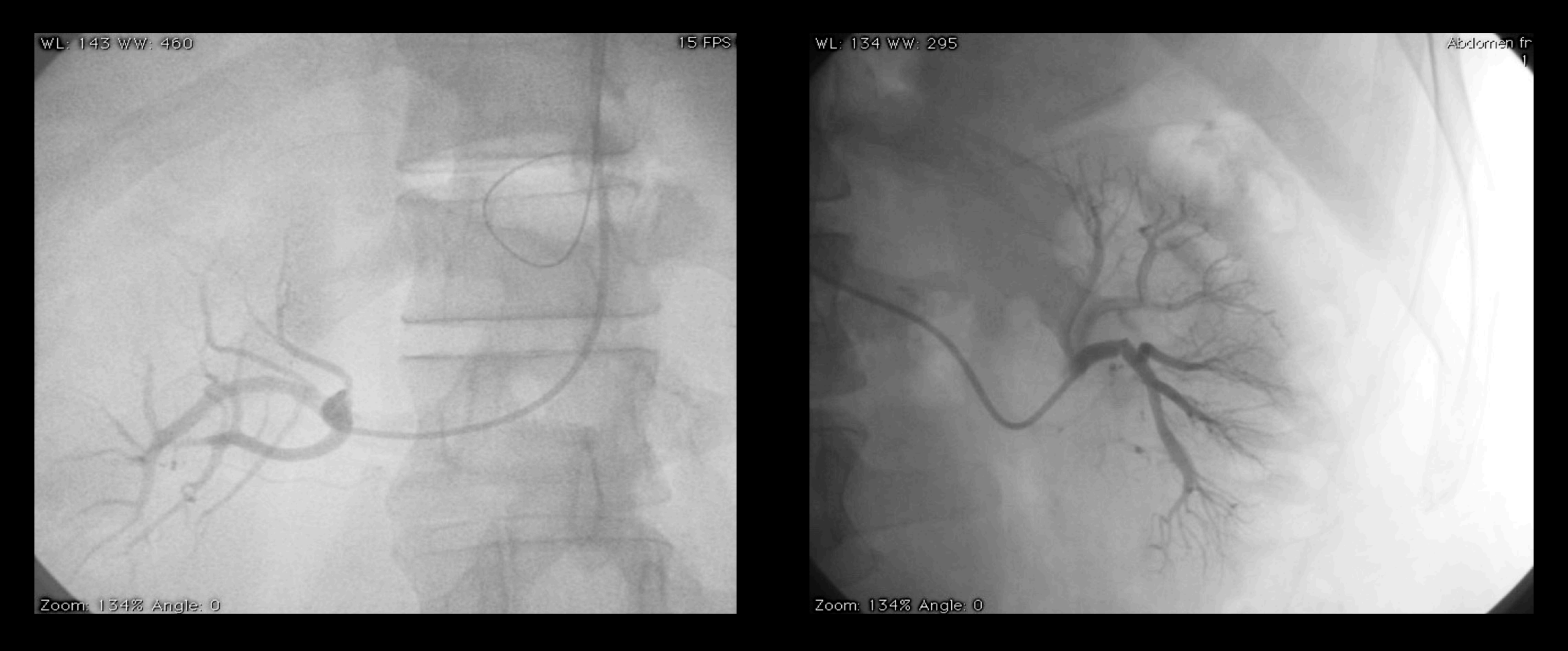
Selective catheterization of both renal arteries for implantation of covered stents.

**Figure 3:**
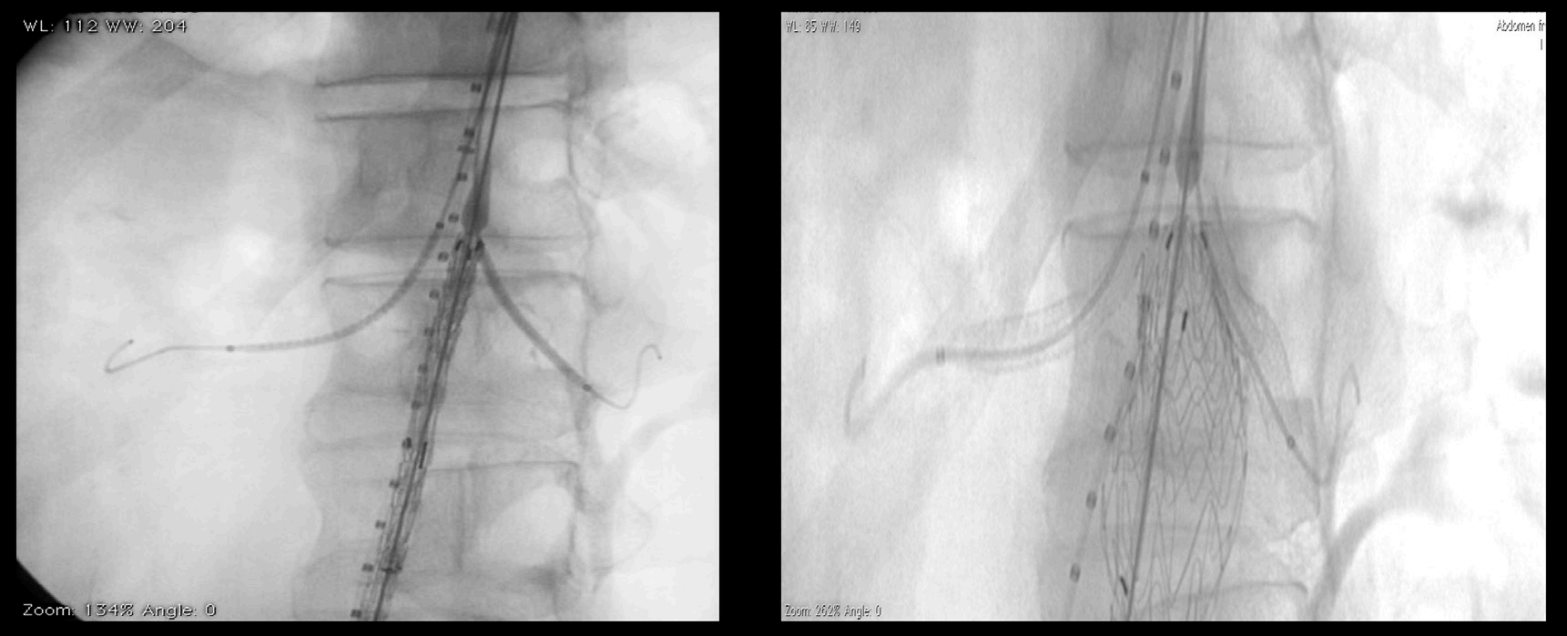
Positioning and release of the endoprosthesis main body and renal stents in the chimney configuration.

Thus a C3 31x150 mm Excluder^®^ (W.L. Gore & Associates, Arizona, USA) bifurcated abdominal aorta endoprosthesis main body and 12x100 mm contralateral endoprosthesis were implanted in conjunction with chimney grafts for both renal arteries using Viabahn^®^ (W.L. Gore & Associates) self-expanding covered stents, 6x50 mm for the right renal artery and 7x50 mm for the left renal artery (Figures 2 and 3).

The procedure was conducted under general anesthesia with antibiotic prophylaxis, followed by dissection of both femoral arteries and the left brachial artery, and ultrasound-guided puncture of the right brachial artery. The femoral accesses were used as routes for deployment of the main body and contralateral endoprostheses and the upper limb accesses were used to implant the renal stents in parallel with the abdominal endoprosthesis. Approximately 120 mL of non-ionic iodinated contrast was used and the total procedure time was approximately 2 hours.

There were no complications during the immediate postoperative period, the patient was transferred from the intensive care unit after 24 hours, already free from abdominal pain, and was discharged from hospital the third day after admission, on double platelet antiaggregation.

**Figure 4:**
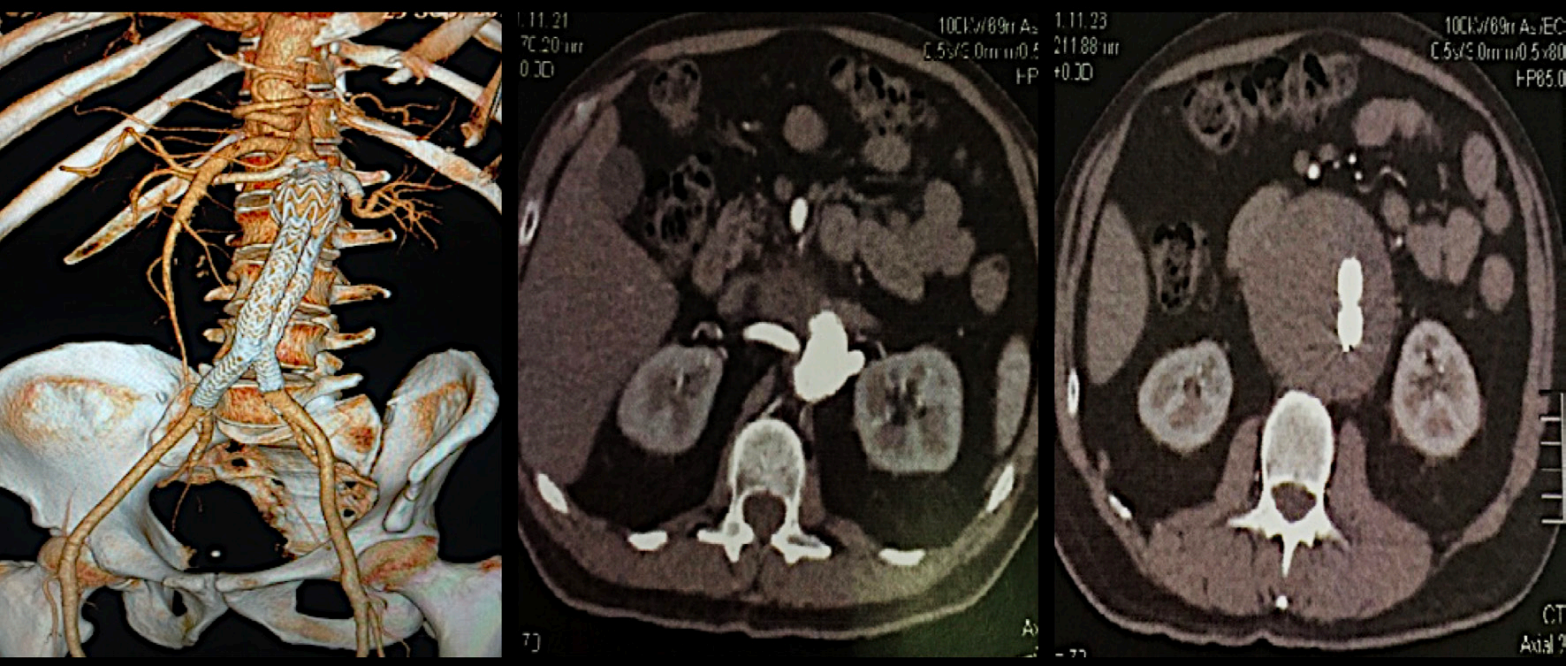
Two-year follow-up angiotomography, with 3D reconstruction and axial images showing exclusion of the aneurysm and preservation of flow in both renal arteries.

The patient was kept in outpatients follow-up for 24 months and underwent serial angiotomography examinations (at 1 month, 3 months, 6 months, 1 year, and 2 years) which showed complete exclusion of the aneurysm, no signs of leaks or stent migration, and patency preserved in both renal arteries (Figure 4).

The patient remains asymptomatic from an abdominal point of view, with renal function preserved and recent follow-up with Duplex vascular echography showed patent renal stents, no signs of restenosis, and no echographic signs of leakage. The aneurysm diameter has reduced by approximately 15% compared to the last control angiotomography.

## DISCUSSION

The EVAR technique with stents in parallel is designed to exclude the aneurysm while maintaining flow through visceral vessels. The technique has proved feasible in selected patients with juxtarenal, pararrenal, or thoracoabdominal aortic aneurysms with good initial success rates.^3,8^

Open “conventional” surgery is not a viable option in many patients of advanced age with significant comorbidities, since suprarenal clamping is necessary, introducing considerably higher risk of perioperative morbidity and mortality.^9^ A study conducted in Edinburgh suggests that 26% of patients admitted with JR-AAA were not considered candidates for open repair.^10^

Studies have been describing treatment with fenestrated devices since the end of the twentieth century^11^ and there has been continuous development since then. Initially, devices were manually fenestrated during the surgical operation by the surgeons, but now preoperative fenestrated customization by the manufacturer (F-EVAR) and OTS devices are available.^12^ However, customized devices require a minimum period of time to allow data analysis, completion of production, sterilization, and delivery. They cannot therefore be used for urgent or emergency repair.

Studies using customized devices have shown clinical success over the short term.^13,14^ However, an anatomic viability study showed that only 63% of JR-AAA were suitable for the fenestrated devices available.^15^

The potential advantages of Ch-EVAR in relation to F-EVAR include reduced complexity, greater availability at smaller centers, and an option for immediate treatment in urgent and emergent situations. Ch-EVAR can be performed without planning and customization of devices in advance, in addition to involving lower cost and shorter training time when compared to F-EVAR.^6^

Current evidence suggests that Ch-EVAR can be performed with the majority of abdominal endoprostheses, although in vitro data indicate that there are differences between the different devices in terms of gutter size and compressive forces on the chimney.^16^ The ideal degree of oversizing remains an unanswered question, but the minimum recommended is greater than 20%.^16-18^

The authors of one systematic review^19^ reported less than 5% incidence of occlusion of chimney stents in the visceral vessels and no significant differences between the different types of covered stent employed. Uncovered stents are also being used as chimney grafts for treatment of JR-AAA with acceptable initial results and further reductions in the cost of the procedure.^20^

Results of the PERICLES multicenter registry were reported in 2015. In that study, 517 patients underwent Ch-EVAR, with a total of 898 chimney grafted branch vessels. Median follow-up was 17 months, and the survival rate in a cohort of high-risk patients was 79%, with a 94% primary patency rate for branches and a 95% secondary patency rate.^21^ In a recent publication,^22^ four specialists from three centers give their opinions on the best endoprosthesis model, with regard to presence or absence of free flow, for use in parallel stenting technique for treatment of aortic aneurysms.^22^

Three authors from two centers recommend using endoprostheses with suprarenal fixation for the following reasons: to avoid occlusion of the branch stent by the covered segment of the endoprosthesis, to achieve better fixation/stabilization of the branch stent, avoiding “toppling” or “collapsing”, and to facilitate repeat catheterization in the future.^22^

The fourth author prefers to use endoprostheses without free flow, of the same model used in the case described here, because of their high conformability and absence of free flow with active fixation that could perforate the parallel grafts, and because they are made from the same material (polytetrafluoroethylene and nitinol) and have similar radial forces as the branch graft.^22^ Robust studies are still needed investigating the impact of endoprosthesis fixation systems on the rates of leaks and patency of visceral branches for parallel stents techniques.

With regard to use of fenestrated stents, Roy et al.^23^ conducted a retrospective study analyzing long term results of 173 patients treated with F-EVAR to repair JR-AAA, finding a hospital mortality rate of 5.2%, and mean survival of 7.1 years. The study concluded that fenestration was associated with a low mortality rate over the long term, but with a significant endovascular reintervention rate.

Oderich et al. published results of a United States multicenter study of treatment of JR-AAA with a fenestrated stent model, concluding that fenestration is safe and effective with low morbidity and mortality in well-chosen cases treated at specialist centers.^24^

A French study published in 2014 did not detect statistically significant differences in short and medium term results between JR-AAA patients treated with Ch-EVAR or F-EVAR.^25^

Li et al.^26^ analyzed the results of endovascular treatment of JR-AAA using the F-EVAR (nine cohort studies) and Ch-EVAR (eight cohort studies) techniques and concluded that both techniques are effective for treatment of JR-AAA, with fenestration as first-choice option and parallel stenting most often used in cases with complex anatomy or emergencies. These data appear to indicate that Ch-EVAR is a safe technique with immediate availability for treatment of complex aneurysms in patients at high risk from conventional surgical treatment, when there is an urgent need for treatment of aneurysmal disease and/or anatomy that is unfavorable for fenestration.

## FINAL COMMENTS

Nowadays, specialists should be accustomed to the techniques and materials available for treatment of juxtarenal aneurysms. This article presented a case of urgent treatment of a JR-AAA using the chimney technique to both renal arteries, with good medium-term results, proving to be a feasible and viable technique. Long-term studies are needed to determine the true role of parallel stents techniques for treatment of aneurysms of the abdominal aorta with challenging anatomy.
