# Supplementary material for: Urgent endovascular treatment of juxtarenal aneurysm using a parallel grafts technique
Source: J Vasc Bras. 2024 Oct 4;23:e20200028. doi: 10.1590/1677-5449.202000282 (PMC11486468; doi:10.1590/1677-5449.202000282)
Supplement: Translation

# Translation: Tratamento endovascular de urgência do aneurisma justarrenal utilizando técnica de stents em paralelo

**DOI:** 10.1590/1677-5449.202000281

**Authors:** Fábio Augusto Cypreste Oliveira, Bruno Campos Coelho, Marina Balestra, Juliana Caetano Barreto Cypreste Oliveira, Maria Cunha Ribeiro Amorelli, Davi Douglas Heckmann, Carlos Eduardo de Sousa Amorelli, Fábio Lemos Campedelli

**Affiliations:** 1 Hospital São Francisco de Assis, Serviço de Cirurgia Vascular e Endovascular, Goiânia, GO, Brasil.; 2 Universidade Federal de Goiás – UFG, Hospital das Clínicas, Angiorradiologia e Cirurgia Endovascular, Goiânia, GO, Brasil.; 3 Universidade Federal de Goiás – UFG, Hospital das Clínicas, Infectologia, Goiânia, GO, Brasil.; 4 Hospital São Francisco de Assis, Hematologia e Hemoterapia, Goiânia, GO, Brasil.; 5 CenterVasc Rio, Rio de Janeiro, RJ, Brasil.

**Keywords:** aneurisma da aorta abdominal, procedimentos endovasculares, stents

## Abstract

O aneurisma da aorta abdominal pode representar um desafio terapêutico em várias condições anatômicas, tornando complexo o seu tratamento endovascular. O aneurisma de aorta abdominal justarrenal (AAA-JR) é definido pela ausência de uma zona proximal de fixação no segmento infrarrenal, e, dessa forma, técnicas são utilizadas para obter um colo adequado para a fixação das endopróteses sem promover a oclusão das artérias renais e dos ramos viscerais. As técnicas de stents em paralelo, fenestração em bancada, customização pela indústria e utilização de endoprótese ramificada de prateleira são utilizadas nos aneurismas abdominais com colo proximal inadequado, porém cada técnica apresenta indicação, limitação e riscos. Neste desafio terapêutico, apresenta-se um caso de tratamento endovascular de urgência de um AAA-JR com a técnica de stents em paralelo com boa evolução a médio prazo e discute-se suas opções terapêuticas.

## INTRODUÇÃO

Desde a sua criação, no início da década de 90, o reparo endovascular do aneurisma da aorta abdominal (EVAR) evoluiu a um nível em que mais de 70% de todos os aneurismas cirúrgicos da aorta abdominal podem ser reparados por essa técnica^1^ . Entretanto, dificuldades com as vias de acesso, as calcificações circunferenciais, as angulações extremas e o colo proximal e/ou distal ausentes ou inadequados podem tornar o EVAR um desafio.

O aneurisma de aorta abdominal justarrenal (AAA-JR) é caracterizado pelo início da dilatação do aneurisma imediatamente abaixo da origem das artérias renais, determinando a ausência de zona proximal de fixação no segmento infrarrenal. Dessa forma, técnicas endovasculares podem ser utilizadas para a obtenção proximal de colo adequado ao selamento proximal sem promover a oclusão dos ramos viscerais da aorta abdominal^2^ .

O implante de stents em paralelo foi inicialmente desenvolvido como uma manobra de resgate para manter a permeabilidade dos ramos da aorta que foram inadvertidamente cobertos durante o EVAR^3^ . As técnicas de stents em paralelo (chaminé/*snorkel* e sanduíche) oferecem uma opção com menor custo quando comparadas aos dispositivos customizados (CMD) ou de prateleira [*off-the-shelf* (OTS)] e com maior facilidade de uso pela disponibilidade, sendo uma importante opção no tratamento de emergência e urgência dos aneurismas da aorta.

A experiência inicial com a técnica de *snorkel* foi publicada em 2003 por Greenberg et al.^4^ , e Lobato e Camacho-Lobato^5^ descreveram a experiência com a técnica de sanduíche em 2012.

Essas técnicas parecem proporcionar uma boa conformabilidade entre a endoprótese principal, o stent visceral e a parede da aorta; no entanto, há um potencial para o surgimento de goteiras entre os dispositivos em paralelo e, consequentemente, um tipo específico de vazamento denominado *gutter leak* ^6^ .

Relatamos, a seguir, um caso de tratamento de urgência de um paciente com AAA-JR, utilizando a técnica de chaminé (Ch-EVAR) como tratamento de escolha.

## PARTE I – SITUAÇÃO CLÍNICA

Paciente masculino, 62 anos, hipertenso e com história de angioplastia coronariana há dois anos, deu entrada com quadro de dor abdominal de forte intensidade com irradiação para dorso, de início súbito e evolução de 24 horas. Ao exame físico, apresentava-se estável hemodinamicamente, com abdome globoso, doloroso à palpação profunda, mas sem sinais de irritação peritoneal e pulsos normais em ambos os membros inferiores.

Foi submetido à tomografia computadorizada de abdome e pelve com contraste que mostrou um AAA-JR com diâmetro máximo de 82 mm e sem outros achados que justificassem a dor abdominal (Figura 1). Devido ao risco iminente de rotura e consequente óbito, havia indicação de tratamento cirúrgico de urgência, e as possíveis técnicas cirúrgicas cabíveis no momento eram:

**Figura 1:** Angiotomografia de abdome e pelve com contraste venoso em cortes axiais evidenciando volumoso aneurisma de aorta justarrenal.

1- Cirurgia convencional com clampeamento suprarrenal;
2- Tratamento endovascular com fenestração em bancada;
3- Tratamento endovascular com chaminé para ambas artérias renais.

## PARTE II – O QUE FOI FEITO

Devido ao paciente apresentar risco cardiológico elevado — ASA III, segundo a *American Society of Anesthesiology* ^7^ — e à necessidade de clampeamento suprarrenal, fato que aumentaria a morbidade cirúrgica e o risco de insuficiência renal, foi optado por realizar o tratamento endovascular com a técnica de stents em paralelo.

A fenestração em bancada também se apresentava como uma opção de abordagem endovascular, porém, aumentaria o tempo cirúrgico, com dificuldade técnica mais elevada. A customização não era factível devido ao tempo necessário para fabricação, esterilização e entrega da endoprótese. Não foi aventada a hipótese de ramificação toracoabdominal, pois havia colo saudável entre a artéria mesentérica superior e o início do aneurisma (28 mm), e o diâmetro da aorta ao nível do tronco celíaco era inferior a 28 mm.

Dessa forma, foi realizado implante de endoprótese bifurcada da aorta abdominal Excluder^®^ (W.L. Gore & Associates, Arizona, USA) C3 31x150 mm corpo principal e endoprótese contralateral 12x100 mm, associada à técnica de chaminé para ambas as artérias renais com stents recobertos autoexpanssíveis Viabahn^®^ (W.L. Gore & Associates), sendo 6x50 mm em artéria renal direita e 7x50 mm em artéria renal esquerda (Figuras 2 e 3).

**Figura 2:** Cateterismo seletivo de ambas artérias renais para implante dos stents revestidos.

**Figura 3:** Posicionamento e liberação da endoprótese corpo principal e stents renais em técnica de chaminé.

O procedimento foi realizado sob anestesia geral com profilaxia antibiótica, seguido de dissecção de ambas artérias femorais e de artéria braquial esquerda, além de punção ecoguiada de artéria braquial direita. Os acessos femorais foram utilizados como via para o implante da endoprótese corpo principal e contralateral, e os acessos nos membros superiores foram utilizados para o implante dos stents renais em paralelo com a endoprótese abdominal. Foi utilizado aproximadamente 120 mL de contraste iodado não-iônico, com um tempo total de procedimento de aproximadamente 2 horas.

A evolução pós-operatória ocorreu de forma favorável e sem intercorrências, com alta da unidade de terapia intensiva em 24 horas, já sem queixa álgica abdominal, e alta hospitalar no terceiro dia de internação hospitalar em uso de dupla antiagregação plaquetária.

O paciente permaneceu em acompanhamento ambulatorial por 24 meses e foi submetido à angiotomografias seriadas (1º mês, 3º mês, 6º mês, 1º ano e 2º ano) com completa exclusão do aneurisma, ausência de sinais de vazamento ou migração das endopróteses e manutenção da perviedade de ambas artérias renais (Figura 4).

**Figura 4:** Angiotomografia de seguimento de 2 anos em reconstrução 3D e cortes axiais mostrando exclusão aneurismática e manutenção do fluxo de ambas artérias renais.

O paciente encontra-se assintomático do ponto de vista abdominal, com função renal preservada e seguimento atual com estudo de ecografia vascular com Doppler mostrando stents renais pérvios, sem sinais de reestenoses e ausência de sinais ecográficos de vazamentos. O diâmetro do aneurisma apresentou uma redução de aproximadamente 15% em comparação com a última angiotomografia de controle realizada.

## DISCUSSÃO

A técnica de stents em paralelos no EVAR tem por objetivo a exclusão aneurismática com manutenção do fluxo dos ramos viscerais. Essa técnica tem se mostrado factível em pacientes selecionados com aneurismas de aorta justarrenal, pararrenal ou toracoabdominal com boas taxas iniciais de sucesso^3,8^ .

A cirurgia aberta, dita convencional, não é uma opção viável para muitos pacientes com idade avançada e comorbidade significativa, visto que, nesses casos, o clampeamento suprarrenal é necessário e acarreta riscos consideravelmente maiores de morbidade e mortalidade perioperatória^9^ . Um estudo de Edimburgo sugeriu que 26% dos pacientes admitidos com AAA-JR não eram considerados candidatos para o reparo aberto^10^ .

Estudos com dispositivos fenestrados foram descritos desde final do século XX^11^ e, desde então, sua evolução é contínua. Inicialmente os dispositivos eram fenestrados durante o ato cirúrgico em bancada manualmente pelos cirurgiões, mas, atualmente, a customização com fenestração pré-operatória pela indústria (F-EVAR) e dispositivos OTS já estão disponíveis^12^ . Os dispositivos CMD necessitam de um período mínimo para análise de dados, término da produção, esterilização e transporte. Dessa forma, dificilmente podem ser utilizados para reparos de urgência ou emergência.

Estudos sobre os dispositivos customizados foram realizados e mostraram sucesso clínico a curto prazo^13,14^ . Entretanto, um estudo de viabilidade anatômica mostrou que apenas 63% dos AAA-JR eram adequados para os dispositivos fenestrados disponíveis^15^ .

As vantagens potenciais do Ch-EVAR em relação ao F-EVAR incluem redução da complexidade, maior disponibilidade em centros menores e uma opção de tratamento imediato no cenário de urgência e emergência. O Ch-EVAR pode ser executado sem o planejamento e a personalização de dispositivos anteriores, além de apresentar custo reduzido e menor tempo de treinamento quando comparado ao F-EVAR^6^ .

Evidências atuais sugerem que o Ch-EVAR possa ser utilizado com a maioria das endopróteses abdominais, embora dados *in vitro* indiquem diferenças entre os vários dispositivos em relação ao tamanho das calhas e às forças de compressão na chaminé^16^ . O “*oversizing*” ideal permanece sem resposta, porém o mínimo recomendado é superior a 20%^16-18^ .

Em uma revisão sistemática^19^ , os autores relataram incidência menor que 5% de oclusão do stent em chaminé nos ramos viscerais e nenhuma diferença significativa entre o tipo do stent recoberto utilizado. Stents não recobertos também estão sendo utilizados como chaminé no tratamento dos AAA-JR com resultados iniciais aceitáveis e redução adicional ao custo do procedimento^20^ .

Os resultados do registro multicêntrico PERICLES foram relatados em 2015. Nesse estudo, 517 pacientes foram submetidos ao Ch-EVAR, apresentando um total de 898 ramos em chaminé. A mediana do tempo de seguimento foi de 17 meses, e a taxa de sobrevida em uma coorte de pacientes de alto risco foi de 79%, com uma taxa de perviedade primária dos ramos de 94% e secundária de 95%^21^ . Em uma recente publicação^22^ , quatro especialistas opinam sobre a melhor opção de modelo de endoprótese, quanto à presença ou não de *free flow* para utilização na técnica de stents em paralelos no tratamento do aneurisma da aorta^22^ .

Três autores de dois centros recomendam o uso de endopróteses com fixação suprarrenal pelos seguintes motivos: evitar a oclusão do stent ponte pelo segmento recoberto da endoprótese e melhor fixação/estabilização do stent ponte, evitando sua queda ou *kinking*, e para facilitar também seu cateterismo futuro^22^ .

O quarto autor recomenda utilizar endoprótese sem *free flow,* do mesmo modelo utilizado no caso aqui descrito, justificando a sua alta conformabilidade, ausência de *free flow* com fixação ativa que poderia perfurar os stents pontes e mesmo material (politetrafluoretileno e nitinol) e a força radial do stent ponte utilizado^22^ . Ainda são necessário estudos robustos sobre o impacto dos sistemas de fixação das endopróteses sobre a taxa de vazamento e a perviedade dos ramos viscerais na técnica de stents em paralelo.

Com relação à utilização de endopróteses fenestradas, Roy et al.^23^ , em um estudo retrospectivo, analisaram a longo prazo 173 pacientes submetidos à F-EVAR para o reparo do AAA-JR e obtiveram uma taxa de mortalidade hospitalar de 5,2%, com uma taxa de sobrevida média de 7,1 anos. Concluiu-se, nesse estudo, que a fenestração apresenta uma baixa taxa de mortalidade a longo prazo, porém com uma taxa de reintervenção endovascular significativa.

Oderich et al., em um estudo multicêntrico americano sobre o tratamento do AAA-JR com um modelo endoprótese fenestrada, concluem que a fenestração é segura e eficaz com baixa morbimortalidade em casos bem selecionados tratados em centros de referência^24^ .

Um estudo francês publicado em 2014 não encontrou diferença estatisticamente significativa nos resultados a curto e médio prazo entre pacientes tratados com Ch-EVAR ou F-EVAR para AAA-JR^25^ .

Li et al.^26^ analisaram os resultados do tratamento endovascular do AAA-JR com as técnicas F-EVAR (nove estudos tipo coorte) e Ch-EVAR (oito estudos tipo coorte) e concluíram que ambas as técnicas são eficazes no tratamento do AAA-JR, tendo a fenestração como opção prioritária e os stents em paralelos sendo mais utilizados em casos de anatomia complexa e urgência. Esses dados parecem indicar que o Ch-EVAR é uma técnica segura e com disponibilidade imediata para o tratamento de aneurismas complexos em pacientes com alto risco ao tratamento cirúrgico convencional, que têm urgência no tratamento da doença aneurismática e/ou anatomia desfavorável à fenestração.

## CONSIDERAÇÕES FINAIS

Atualmente, os especialistas devem estar habituados às técnicas e aos materiais disponíveis ao tratamento do aneurisma justarrenal. No relato, foi apresentado um caso de tratamento de urgência do AAA-JR, utilizando a técnica de chaminé para ambas as artérias renais, que apresentou bom resultado a médio prazo e mostrou-se como uma opção factível e viável. Estudos a longo prazo são necessários para determinar o real papel da técnica de stents em paralelo no tratamento dos aneurismas da aorta abdominal com anatomia desafiadora.
